# Fibroblast Growth Factor Receptors as Novel Therapeutic Targets in SNF5-Deleted Malignant Rhabdoid Tumors

**DOI:** 10.1371/journal.pone.0077652

**Published:** 2013-10-30

**Authors:** Simon Wöhrle, Andreas Weiss, Moriko Ito, Audrey Kauffmann, Masato Murakami, Zainab Jagani, Anne Thuery, Beatrice Bauer-Probst, Flavia Reimann, Christelle Stamm, Astrid Pornon, Vincent Romanet, Vito Guagnano, Thomas Brümmendorf, William R. Sellers, Francesco Hofmann, Charles W. M. Roberts, Diana Graus Porta

**Affiliations:** 1 Novartis Institutes for BioMedical Research, Basel, Switzerland; 2 Novartis Institutes for BioMedical Research, Cambridge, Massachusetts, United States of America; 3 Department of Pediatric Oncology, Dana-Farber Cancer Institute, Division of Hematology/Oncology, Children’s Hospital Boston, Boston, Massachusetts, United States of America; CCR, National Cancer Institute, NIH, United States of America

## Abstract

Malignant rhabdoid tumors (MRTs) are aggressive pediatric cancers arising in brain, kidney and soft tissues, which are characterized by loss of the tumor suppressor SNF5/SMARCB1. MRTs are poorly responsive to chemotherapy and thus a high unmet clinical need exists for novel therapies for MRT patients. SNF5 is a core subunit of the SWI/SNF chromatin remodeling complex which affects gene expression by nucleosome remodeling. Here, we report that loss of SNF5 function correlates with increased expression of fibroblast growth factor receptors (FGFRs) in MRT cell lines and primary tumors and that re-expression of SNF5 in MRT cells causes a marked repression of FGFR expression. Conversely, siRNA-mediated impairment of SWI/SNF function leads to elevated levels of FGFR2 in human fibroblasts. In vivo, treatment with NVP-BGJ398, a selective FGFR inhibitor, blocks progression of a murine MRT model. Hence, we identify FGFR signaling as an aberrantly activated oncogenic pathway in MRTs and propose pharmacological inhibition of FGFRs as a potential novel clinical therapy for MRTs.

## Introduction

Malignant rhabdoid tumors (MRTs) are very aggressive pediatric tumors arising from kidney or extra-renal sites such as brain or soft tissues [1], [2]. MRTs are characterized by loss of function of the tumor suppressor SNF5 (also known as SMARCB1, INI1 or BAF47) due to inactivating mutations or deletions of the *SNF5* gene [3]–[5]. SNF5 is a core component of the SWI/SNF chromatin remodeling complex, which mediates nucleosome repositioning along the DNA in an ATP-dependent fashion [6]. By rendering the genome more or less accessible for the transcriptional regulatory machinery, the SWI/SNF complex has both repressive and activating functions in the regulation of gene expression [7]–[9]. SNF5 does not contain any DNA-binding motifs, but has been shown to form manifold interactions with transcription factors and DNA-regulatory proteins, such as GLI1, c-MYC, MLL/ALL-1, GADD43 and p53 [10]–[14] and has been implicated in the recruitment of the SWI/SNF complex to specific target gene promoters [15]. In particular, SNF5 has a critical function in cell cycle control and affects the pRb tumor suppressor pathway by inducing expression of p16^INK4A^ and repression of cyclin D1 [16]–[18]. Thus, abrogation of SNF5 function leads to hyperphosphorylation of pRb and E2F-mediated cell cycle activation. In addition, inactivation of SNF5 results in the upregulation of multiple oncogenic pathways, such as Hedgehog and Aurora A signaling and the induction of the Polycomb gene *EZH2* [14], [19], [20]. However, to date no targeted therapy exists to specifically inhibit aberrantly activated oncogenic signaling in MRTs and current chemotherapeutic approaches are largely ineffective [21], [22]. We therefore aimed to identify novel and potentially druggable pathways contributing to the oncogenicity of MRTs. To this end, we have utilized the Cancer Cell Line Encyclopedia (CCLE), a collection of almost 1000 cancer cell lines of multiple tumor types comprehensively annotated in terms of genome-scale mRNA expression, gene copy number alterations and gene mutations (23), and for which pharmacological profiles for over 2000 compounds with defined modes of action (MoAs) have been generated. By means of an unbiased computational approach designed to identify chemical vulnerabilities that are selectively active in a defined subset of cell lines, we have found that FGFR inhibitors are significantly more active in MRT cell lines compared to the rest of soft tissue sarcomas. In particular, MRT lines showed sensitivity to NVP-BGJ398, a novel and highly selective pan-specific FGFR inhibitor currently in Phase I clinical trials for cancer indications [23], [24].

The FGF/FGFR signaling family consists of 18 secreted growth factors that act as ligands for four receptor tyrosine kinases, FGFR1, FGFR2, FGFR3, FGFR4, and which exert important functions during development, but also in adult tissues, e. g. in angiogenesis and metabolism [25]. Moreover, de-regulated FGF/FGFR activity due to genetic alterations occurring in various members of this family has been linked to several diseases including cancer.

Here, we identified and validated FGFR-dependency in MRT models and found that SNF5-deficiency correlates with elevated expression of FGFR1 or FGFR2. We describe a novel regulatory function of SNF5 in the transcriptional regulation of FGFRs and demonstrate that SNF5 is directly recruited to the FGFR2 locus. In addition, we show that pharmacological inhibition of FGFRs using NVP-BGJ398 impairs growth of a mouse MRT allograft model *in vivo*, thus highlighting FGFR inhibition as a potential novel therapeutic option for the treatment of MRTs.

## Materials and Methods

### Cell Lines

293FT cells (#R700-07, Invitrogen) were cultured in 500 ml DMEM with 10% FCS (HyClone), 1 mM sodium pyruvate (Amimed), MEM non-essential amino acids (GIBCO) and 2 mM L-glutamine (Amimed). All other cell lines were obtained from ATCC. A204 (#HTB-82), G401 (3CRL-1441) and G402 (#CRL-1440) were maintained in McCoy’s 5A medium (Amimed) containing 10% FCS and 2 mM L-glutamine. BJ cells (#CRL-2522) were maintained in MEM-EBS (Amimed) with 15% FCS, 1 mM sodium pyruvate, MEM non-essential amino acids, 2 mM L-glutamine and antibiotics. SKLMS1 (#HTB-88) and SKUT1 (#HTB-114) were maintained in EMEM (Amimed) with 10% FCS, 1 mM sodium pyruvate and 2 mM L-glutamine.

### Global Compound Selectivity Analysis

To identify compounds which are selectively responsive to MRT cell lines as compared to non-MRT sarcoma cell lines from the CCLE, we used the high-throughput cell line profiling described below. First, each compound sensitivity metric (Crossing Point, Inflection Point, or Activity Area) is log2 transformed and a selectivity score is computed by multiplying the metric Z-score by the absolute value of the metric. We then realize a Wilcoxon test opposing the two groups of cell lines and correct for multiple testing using Benjamini and Hochberg false discovery rate (FDR). We then proceed to an enrichment analysis by the compounds mode of action (MoA) or main target. To this end, we perform a Fisher exact test with compounds considered as significantly differentially responsive when the FDR is below 0.25. The cut-off for the FDR is lenient as this is used for enrichment purposes. Finally, we correct the p-values obtained from enrichment analysis for multiple testing and we consider a significantly enriched MoA when the FDR is below 0.05.

### Gene Expression Analysis of Primary Sarcoma Samples

Primary sarcoma expression data was analyzed from publicly available gene expression profiling datasets (File S2). Probesets analyzed were 211535_s_at for FGFR1, 208228_s_at for FGFR2 and 212167_s_at for SNF5. Data was visualized using Spotfire (TIBCO).

### Immunoblot Analysis

Cells were plated in 6 cm dishes at an initial density of 1.2×10^6^ per dish. Lysates were prepared with M-PER lysis buffer (Pierce) supplemented with Complete protease inhibitor cocktail and PhosSTOP phosphatase inhibitor cocktail tablets (Roche). Cellular lysates were separated by SDS-PAGE, and transferred to PVDF membranes. Proteins were visualized using antibodies to total-ERK1/2 (#9102, Cell Signaling), phospho-ERK1/2 (#9101, Cell Signaling), phospho-Fibroblast growth factor receptor substrate 2 (phospho-FRS2) (#3861, Cell Signaling), SNF5 (#A301-087A, Bethyl), FGFR1 ((#sc-57132, Santa Cruz), FGFR2 ((#sc-122, Santa Cruz), appropriate horseradish peroxidase-labeled secondary antibodies (Amersham and Jackson ImmunoResearch) and a chemiluminescence detection reagent (Pierce). Anti-β-tubulin (T4026, Sigma) or anti-α-actinin ((#05–384, Millipore) was used as a loading control.

### RNA Purification and Quantitative Real-time PCR (qPCR)

RNA was purified with the RNeasy Mini kit (Qiagen). Random hexamer primed cDNA was synthesized with 0.5–2 µg RNA and MultiScribe MuLV reverse transcriptase (Applied Biosystems). Quantitative real-time PCR was performed in an iQ5 Real-Time PCR Detection System (BioRad) using an iQ SYBR Green Supermix (#170–8882, Bio-Rad) and an equivalent of 40 or 80 ng RNA of each sample. The data were normalized to Gapdh expression. Primer sequences used are f-CCAAGGTCATCCATGACAAC and r-AGAGGCAGGGATGATGTTCT for *GAPDH*, f-GGGACATTCACCACATCGACTA and r-GGGTGCCATCCACTTCACA for *FGFR1*, f-TGAAGGAAGGACACAGAATGGA and r-GCCAACAGTCCCTCATCATCA for *FGFR2*, f-GTGATCCATGAGAACGCATC and r-TCAGGCGTCATCAACTTCTC for *SNF5*, f-CTCCGAGAAGGACAAGAAGG and r-TGCATGATGGTGTTCATCAG for BRG1.

### Chromatin Immunoprecipitation

Chromatin immunoprecipitation (ChIP) was performed using the SimpleChIP Enzymatic Chromatin IP Kit (#9003, Cell Signaling). BJ cells were grown to 80% density in 15 cm tissue culture dishes, medium was removed and cells were washed once with PBS before fixation with 2 mM disuccinimidyl glutarate (#20593, Pierce) in PBS for 45 min. Plates were rinsed twice with PBS and 1% formaldehyde in PBS was added for 10 min. Chromatin was prepared as described by the manufacturer and 10 µg input DNA was used for each IP. Antibodies used include rabbit anti-SNF5 (#A301-087A, Bethyl) and normal rabbit immunoglobulin G (IgG) (#2729, Cell Signaling). IP and DNA purification was performed as described by the manufacturer and 5 µl of the immunoprecipitated DNA and 5% of the reference material were used as templates for qPCR using an iQ SYBR Green Supermix. Primers sequences used to monitor enrichment of FGFR2 promoter DNA were f-AGGCTGAAAGCACACAGTTG and r-CCTGGTCTCAGTGGGAGTTT (−*9133*), f-TGCGAAGAAAAGAGACCTCA and r-AAGGGCAGAAAAGCCAGTAA (−*2420*), f- AACTTAAGCACGGCTGCTC and r- CAACTGCACACCAAGCTGTA (−*1021*), f- AACATTTCCAAGTGGCTTCC and r-ACTTTAAAATGCGCCTGCTT (−*462*), f-CTCTGAGCCTTCGCAACTC and r- AAGAAAGGACTCAGGCTTGG (*+207*), f- AGGACCACTCTTCTGCGTTT and r- GATTACCTTGAATGGCAACG (*+397*), f-TCTGTGGCTGCATAGGTGAT and r-TAGCAGAGGCAGAACTTCCA (*+639*), f-CGAACTGGACCGACTTTTTC and r-AATGAGCGCGCAAGTTAGA (*+1108*), f-TGCTTTTGTAGTTGCCCTTG and r-CTCAGATACGTGCAGCCACT (*+4118*). Numbers indicate primer location relative to transcriptional start site. Specificity of DNA enrichment was controlled using the EpiTect ChIP qPCR Primer Assay Human IGX1A (#GPH100001C(-)01A, Qiagen). The CDKN1A gene served as a positive control for ChIP. Primer sequences used were f- AGCAGGCTGTGGCTCTGATT and r-CAAAATAGCCACCAGCCTCTTCT.

### High-throughput Cell Line Profiling

Cell lines were obtained from ATCC, DSMZ and HSSRB and cultured in RPMI or DMEM plus 10% FBS (Invitrogen) at 37°C 5% CO_2_ using automated processing. A detailed description of the high-throughput cell viability assays can be found in reference [26]. In brief, assays were automated and performed with an ultra-high throughput screening system. Cell lines were dispensed into tissue culture treated 1536 well plates. Compound dilutions were transferred to the cells resulting in a final concentration range of 30 µM to 2.5 nM and a uniform DMSO concentration of 0.4%. 72 to 84 hours cell growth was analyzed with Cell Titer Glo assay (Promega). All dose-response data was reduced to a fitted model using a propriety decision tree methodology that is based on the NIH/NCGC assay guidelines. Parameters derived from the models include: IP, the Inflection Point of the curve; Crossing Point (CP), the concentration where the fitted curve crosses −50%; Amax, which is the maximal activity value reached within a model and Activity Area (AA) as the area under the dose response curve and is a summary metric of efficacy and potency. Data from the high-throughput proliferation assays with NVP-BGJ398 is listed in Supplemental File S1 in reference [27] and File S1 of the current manuscript.

### SNF5 Re-expression in MRT Lines

Retroviral supernatants were collected from 293FT cells transfected with pBabe/FL-Ini1 [28] and pCL-10A1 packaging vector (Imgenex). For re-expression of SNF5 MRT lines were plated in 6-well plates at initial densities of 1.2 to 2.4×10^5^ cells/well and retroviral supernatant was applied the following day twice for 4 h. Puromycin selection was initiated at 48 h post retroviral transduction and RNA and protein lysates or RNA were prepared at day 3 post selection.

### siRNA Transfection

Cells were plated in 6-well plates at initial densities of 10^5^ cells/well one day before transfection with siRNA at concentrations of 50 nM using Lipofectamine RNAiMAX transfection reagent (Invitrogen) as described by the manufacturer. Oligos were obtained from Qiagen and included assays SI00726810, SI04237457, SI04297762 for targeting of *SNF5* and SI00047579, SI03098998, SI00047586 for *BRG1*. AllStars Negative Control oligos (1027280) were used as nonsilencing siRNA control. RNA or protein lysates were prepared at 72 h post transfection.

### Allograft Studies

The experimental procedures involving animal studies strictly adhered to the Association for Assessment and Accreditation of Laboratory Animal Care International (AAALAC) guidelines as published in the “Guide for the Care and Use of Laboratory Animals”, and Novartis Corporate Animal Welfare policies. All animal experiments were fully approved by the Kantonales Veterinäramt Basel-Stadt and were conducted in accordance with the Eidgenössisches Tierschutzgesetz and the Eidgenössische Tierschutzverordnung.

Primary mouse MRT samples were derived from *SNF5*-heterozygous mice, which develop tumors upon spontaneous inactivation of the functional allele [29], [30]. Tumor samples were propagated in athymic nude mice for a maximum of three passages before subcutaneous transplantation into nude mice for efficacy studies. Treatment with NVP-BGJ398 or vehicle control started when average tumor size was at least 100 mm^3^ and tumor volumes were monitored at the indicated times over the course of treatment. At the end of the treatment, mice were euthanized by CO_2_ inhalation. Tumors were excised and snap-frozen in liquid nitrogen or immediately fixed in 4% formaldehyde for 24 h. Frozen tissues were pulverized using a MM200 swing mill (RETSCH) and protein lysates were prepared from tumor powder for further immunoblot analysis. For IHC analysis fixed tumor samples were dehydrated using the TPC Tissue Processing System (MEDITE) before embedding in paraffin. IHC staining using phospho-ERK1/2 antibodies (#4370, Cell Signaling, dilution 1∶200) was performed on 2.5 µm sections on an on automated slide processor (Ventana).

### Statistical Analysis

All data shown represent mean ± standard error of the mean (SEM). Statistical analyses were performed using Student’s t tests (two-tailed). A significance level of p<0.05 is indicated by an asterisk (*).

Material and Methods referring to Figures S1, S2, S3, and S4 are outlined in the Material and Methods S1 section.

## Results

### Malignant Rhabdoid Tumor Lines are Dependent on FGFR Signaling for Proliferation

**Figure 1:**
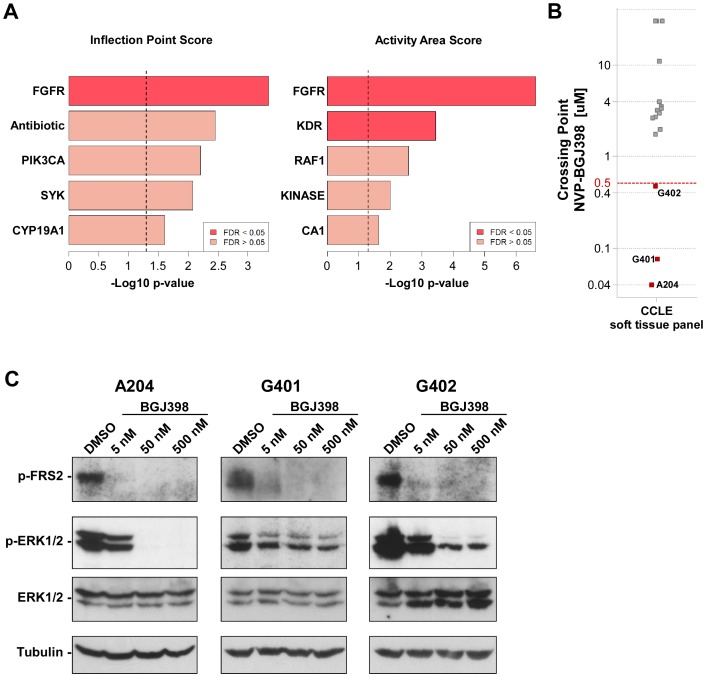
MRT cell lines are sensitive to pharmacological FGFR inhibition. (A) Global compound selectivity analysis of MRT lines. Comparison of the three MRT lines A204, G401 and G402 versus other soft tissue cancer lines from the CCLE with regards to sensitivity to a panel of approximately 2000 compounds with defined target specificity. Shown are the top 5 enriched target in MRTs according to Activity Area and Inflection Point scores. FDR, false discovery rate. (B) Sensitivity towards the FGFR inhibitor NVP-BGJ398 among soft tissue cancer lines. A cut-off value of 500 nM was used to determine NVP-BGJ398 sensitivity based on Crossing Point values from high-throughput cell proliferation assays. (C) Immunoblot analysis of p-FRS2 and p-ERK1/2 in MRT lines treated with DMSO or NVP-BGJ398 for 40 min as indicated. Total ERK1/2 and β-Tubulin expression was used to monitor equal loading.

To identify pathways conferring dependence in MRTs, we analyzed the pharmacological profiling data generated for over 2000 chemical entities with characterized MoAs across the Cancer Cell Line Encyclopedia (CCLE) project. Specifically, we performed an unbiased selectivity analysis of small molecule inhibitors among a panel of 17 soft tissue cancer lines present in the CCLE, including three lines of the MRT subtype - G401, G402 and A204. To this end, sensitivity response to 2000 compounds, grouped by their MoA (or main target), measured in high-throughput proliferation assays was compared between the MRT and the non-MRT sarcomas within the CCLE. As a measure of compound efficacy and potency, we derived three parameters, Activity Area (AA), Crossing Point (CP) and Inflection Point (IP) as described in Materials and Methods section. Using AA and IP scores we identified FGFR as the MoA with the strongest enrichment in the MRT subset (Fig. 1A). FGFR was the only target significantly enriched in MRTs with an FDR below the cut-off value of 0.05 when using both IP and AA. KDR/VEGFR was also a significantly enriched MoA when using AA, but not when using IP indicating more limited overall efficacy of the KDR compounds in MRTs as compared to non-MRTs. Interestingly, when using CP as an alternative read-out for compound sensitivity, FGFRs also scored as the most relevant targets in MRTs, even though not passing the cut-off of FDR <0.05 (data not shown).

We next analyzed the response data of the soft tissue sarcoma cell lines to NVP-BGJ398, our specific FGFR inhibitor in clinical trials, which was included in the selectivity analysis described above. Consistent with the previous CCLE study performed for this compound [24], only cancer cell lines whose proliferation was inhibited with IC_50_ values <500 nM were classified as sensitive. In line with the selectivity analysis, we found that among the 17 soft tissue cancer cell lines, only the three rhabdoid tumor cell lines were sensitive to NVP-BGJ398 treatment. (Fig. 1B and File S1). In addition to the high-throughput analysis, we tested FGFR-dependence of the three MRT lines using NVP-BGJ398 in manual colorimetric proliferation assays and found that NVP-BGJ398 impaired proliferation of G402 cells with an IC_50_ of 249.9 nM. In G401 and A204 cells, treatment with NVP-BGJ398 induced a strong growth inhibition with IC_50_ values of 15.0 nM and 40.5 nM, respectively (Fig. S1A–C). In line with these results, we observed constitutive phosphorylation of the immediate FGFR downstream target FRS2 in all three MRT lines, which was blocked in the presence of NVP-BGJ398 (Fig. 1C). In addition, FGFR inhibition also affected downstream MAPK signaling in the MRT lines, as indicated by a reduction of ERK1/2 phosphorylation in response to NVP-BGJ398 treatment.

**Figure 2:**
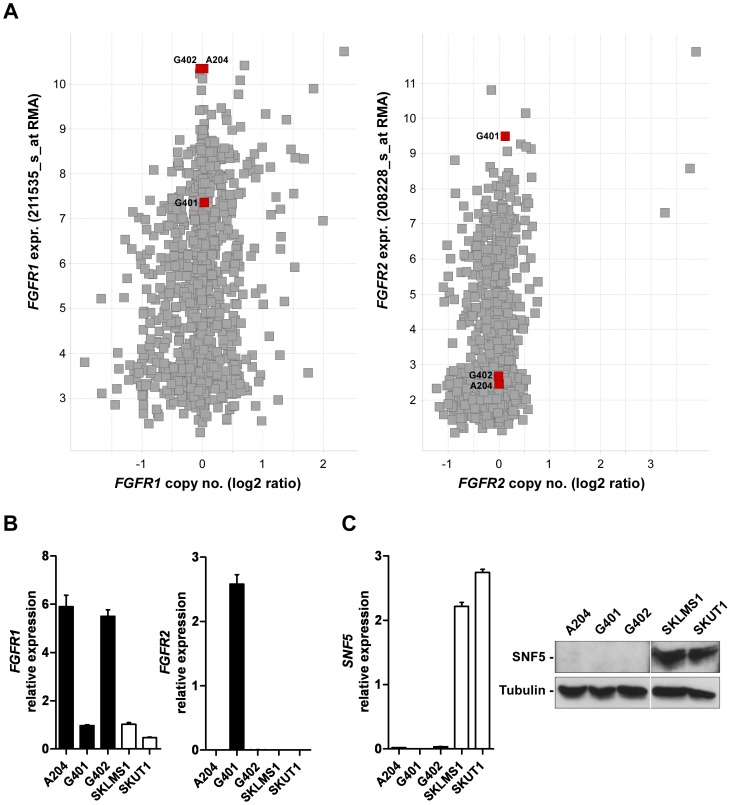
FGFR expression levels of the MRT cell lines A204, G401 and G402. (A) Scatter plot showing expression and copy number levels for *FGFR1* (left panel) and *FGFR2* (right panel) within the CCLE. MRT lines A204, G401 and G402 are indicated in red. (B) Quantitative RT-PCR (qRT-PCR) analysis of *FGFR1* and *FGFR2* mRNA expression in MRT cell lines and soft tissue cancer lines SKLMS1 and SKUT1. Expression values are given as average with standard errors of the mean (SEM) (n≥3) with respect to *GAPDH* mRNA levels (arbitrarily set as 100). (C) qRT-PCR and immunoblot analysis of SNF5-deficiency in MRT lines. SKLMS1 and SKUT1 cells were used as positive controls for SNF5 expression. *SNF5* mRNA expression is given as average with SEM (n≥3) with respect to *GAPDH* mRNA levels (arbitrarily set as 100). β-Tubulin expression was used to monitor equal loading.

To investigate the cause for constitutive FGFR pathway activation and FGFR-dependency for growth observed in the MRT cell lines, we analyzed the genomic data for the FGFR family members generated for these cell lines. The mutational status of the *FGFR* genes was determined by both exon capture sequencing and PCR-based sequencing and revealed absence of sequence variations as compared to the wild type reference sequence (data not shown). However, analysis of Affymetrix transcript expression data revealed that within the CCLE dataset, A204 and G402 were among the lines with the highest expression levels of *FGFR1* and the G401 line displayed high levels of *FGFR2* (Fig. 2A), while all three lines showed low or moderate levels of *FGFR3* and *FGFR4* (Fig. S2). In addition, by means of qRT-PCR we confirmed the elevated levels of *FGFR1* or *FGFR2* transcript in the MRT cells in comparison to two non-FGFR-dependent sarcoma lines, SK-LMS1 and SK-UT-1 (Fig. 2B). Notably, the high expression levels of *FGFR1* or *FGFR2* were observed in the absence of gene copy number gains as assessed using the SNP6.0 Affymetrix platform, suggesting that transcriptional mechanisms may be responsible for the elevated FGFR expression (Fig. 2A). Taken together, this data indicates that MRT lines aberrantly activate and depend on FGFR signaling.

### Re-expression of SNF5 in MRT Lines Abrogates FGFR Expression

**Figure 3:**
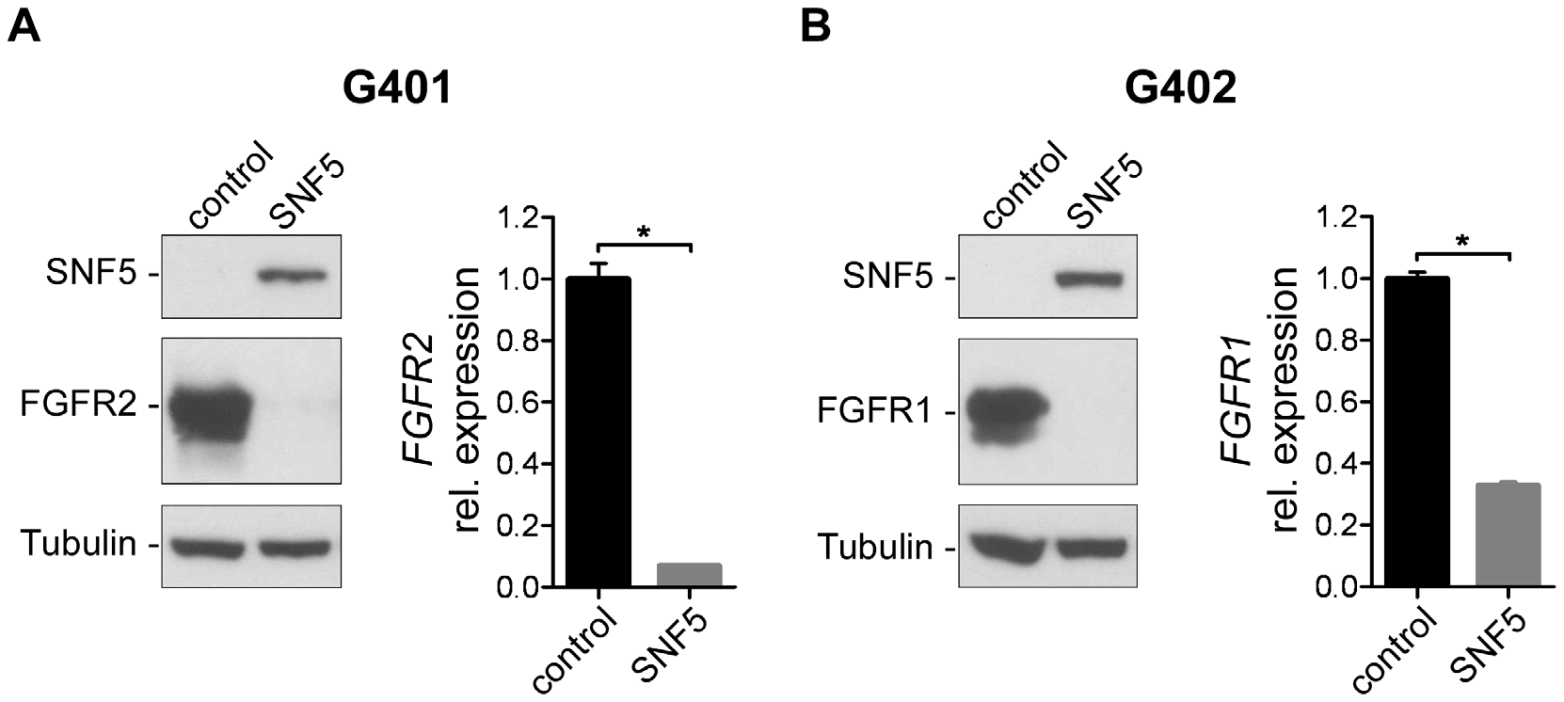
Re-expression of SNF5 in MRT lines abrogates FGFR expression. (A) Immunoblot analysis (left panel) of FGFR2 protein expression in G401 cells five days post retroviral transduction of SNF5. β-Tubulin expression was used to monitor equal loading. *FGFR2* mRNA levels (right panel) were determined by qRT-PCR. Expression is shown as relative levels to control infected cells. Data are given as average with SEM (n = 3). *FGFR2* mRNA expression values were normalized to *GAPDH* mRNA copies. Data were compared by unpaired Student’s t test; *p<0.05. (B) Analysis of FGFR1 protein and mRNA expression in G402 cells upon re-expression of SNF5 as described in (A).

MRTs are characterized by loss of the tumor suppressor SNF5, a component of the SWI/SNF complex, which we confirmed in the three rhabdoid cell lines A204, G401 and G402 (Fig. 2C). Since the SWI/SNF complex has been shown to have both, repressive and activating functions on gene expression, we hypothesized that loss of SNF5 function underlay the elevated FGFR expression observed in MRT lines. To test this hypothesis, we re-introduced SNF5 in the MRT lines G401 and G402 by means of retroviral transduction. In both G401 and G402 we found that re-expression of SNF5 abrogated elevated protein expression of FGFR2 and FGFR1, respectively, which was paralleled by a decrease in mRNA levels (Fig. 3A and B). Similarly, we observed a striking repression of FGFR1 levels upon re-expression of SNF5 in the MRT line A204 (Fig. S3A). To investigate whether the functional relationship between SNF5 and FGFR may be present in other lineages beyond rhabdoid tumors we interrogated the CCLE to identify additional cell lines which would be characterized by loss of SNF5. Interestingly, among seven SNF5-deficient non-MRT lines (Fig. S3D), two of them, the rhabdomyosarcoma line KYM1 and the lung adenocarcinoma line HLC1, also displayed high levels of *FGFR1* and *FGFR2* transcripts, respectively (Fig. S3E and F), which were abrogated upon reconstitution of SNF5 (Fig. S3B and C).

Re-expression of SNF5 causes cell cycle arrest associated with activation of p16^INK4A^ and inhibition of the CDK4/RB/E2F pathway [16], [17]. To rule out a non-specific effect on FGFR expression in response to the cell cycle arrest caused by forced SNF5 re-expression in the MRT lines, we treated the MRT lines A204 and G402 with the cell cycle inhibitor Staurosporine. While Staurosporine treatment strongly impaired proliferation, no effects on FGFR expression were observed in both cell lines (data not shown). In summary, this data illustrates that SNF5 negatively affects expression of FGFR1 and FGFR2 and indicates that MRT cell lines express elevated levels of FGFRs as a consequence of SNF5 loss of function.

### SNF5 Loss of Function Leads to Increased Expression of FGFR2 in Normal Human Fibroblasts

**Figure 4:**
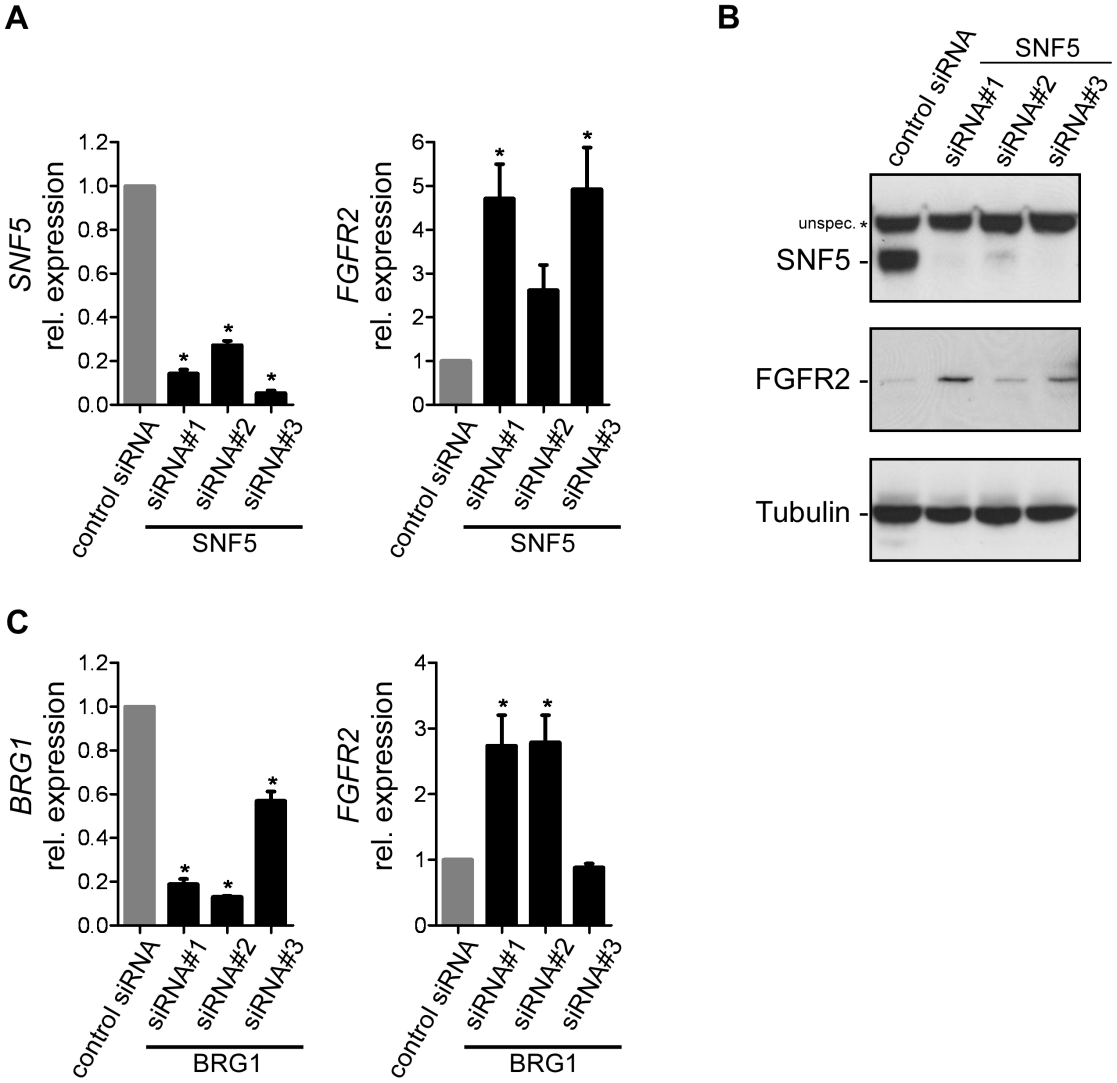
SNF5 loss of function induces FGFR2 expression in human fibroblasts. (A) Effect of siRNA-mediated knockdown of SNF5 on FGFR2 expression in BJ cells. *SNF5* and *FGFR2* expression levels were analyzed by qRT-PCR at 72 h post siRNA transfection. Expression is shown as relative levels to cells transfected with non-targeting control siRNA and is given as average with SEM (n≥3). *E*xpression values were normalized to *GAPDH* mRNA copies. (B) Immunoblot analysis of FGFR2 expression upon knockdown of SNF5 in BJ cells as described in (A). β-Tubulin expression was used to monitor equal loading. (C) Effect of siRNA-mediated knockdown of BRG1 on FGFR2 expression in BJ cells as described in (A).

In addition to the re-expression studies in SNF5-deficient cell lines, we also evaluated whether inactivation of SNF5 would affect FGFR expression in cell lines with an intact SWI/SNF complex. We tested the effect of siRNA-mediated SNF5-knockdown in a panel of non-MRT sarcoma cell lines with low or moderate *FGFR1* and *FGFR2* levels, as well as in BJ cells, a non-immortalized human fibroblast line, previously reported to have a functional SWI/SNF complex [19]. While we did not observe any effects on FGFR expression upon SNF5-knockdown in the sarcoma cell lines tested, we found that in BJ cells knockdown of SNF5 led to increased mRNA levels of *FGFR2* (Fig. 4A). The transcriptional induction of FGFR2 upon SNF5 knockdown was paralleled by an increase in FGFR2 protein levels (Fig. 4B). Since SNF5 is a core component of the SWI/SNF chromatin remodeling complex, we tested whether inhibition of SWI/SNF function by knockdown of the ATPase core subunit BRG1 would similarly affect FGFR2 expression in BJ cells and found that loss of BRG1 function also led to induction of FGFR2 (Fig. 4C). These results demonstrate that SNF5 represses FGFR2 transcription in a SWI/SNF-dependent fashion in human fibroblasts.

### SNF5 is Localized to the FGFR2 Promoter in Human Fibroblasts

**Figure 5:**
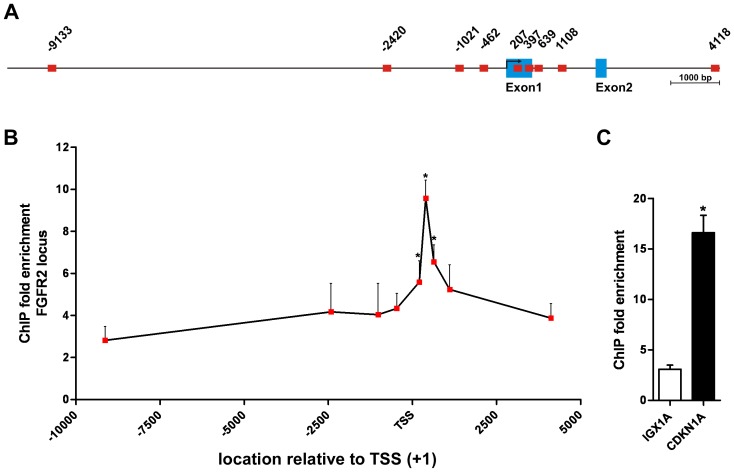
SNF5 is recruited to the FGFR2 promoter in BJ cells. (A) Schematic overview of the human FGFR2 promoter. Amplicons of primer pairs used for ChIP are shown as red squares and location is indicated relative to the transcriptional start site (TSS, +1). Exons are shown as blue boxes. (B) FGFR2 promoter occupancy by SNF5 in BJ cells. Fold enrichment from chromatin immunoprecititaions (ChIP) with a SNF5-specific antibody compared to an IgG control was analyzed by qPCR using the primer pairs indicated in (A). (C) Fold enrichment of the negative control locus IGX1A and the promoter region of the known SNF5 target gene CDKN1A. Fold enrichment is given as average with SEM (n≥3). Data were compared by unpaired Student’s t test with respect to the fold enrichment of the IGX1A locus; *p<0.05.

SNF5 has been shown to be directly recruited to target gene promoters and to suppress gene transcription by modification of the adjacent chromatin structure [14], [19], [20], [31]. To determine whether FGFR expression is controlled by SNF5 in a similar fashion we analyzed SNF5 localization to the FGFR2 promoter in BJ cells by chromatin immunoprecipitation (ChIP). To this end, we designed a series of primer pairs spanning a region from approximately 10 kb upstream to 4 kb downstream of the transcriptional start site (TSS) in the human FGFR2 promoter (Fig. 5A) and identified occupancy of the FGFR2 promoter by SNF5 with a focal peak binding site closely following the TTS (Fig. 5B). At this site, we found an approximately 10-fold enrichment of ChIP-DNA compared to the basal enrichment levels present at distal sites and the negative control locus IGX1A, and similar to the fold enrichment observed for the known SNF5 target gene CDKN1A (Fig. 5C). This indicates that SNF5 exerts its repressive function on FGFR2 expression via a direct interaction with the FGFR2 promoter locus.

### Inhibition of FGFR Signaling Impairs MRT Growth In Vivo

**Figure 6:**
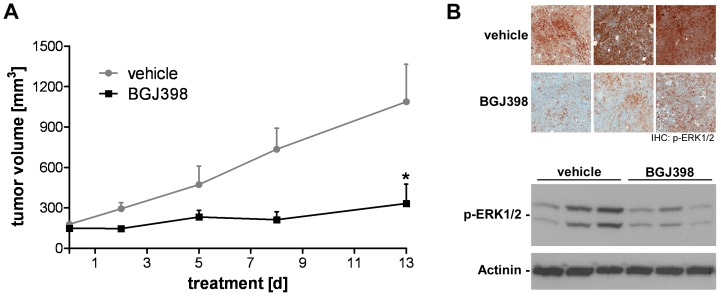
FGFR inhibition with NVP-BGJ398 impairs MRT growth *in vivo*. (A) *In vivo* efficacy of NVP-BGJ398 in a primary mouse MRT allograft model. MRT bearing nude mice received vehicle or NVP-BGJ398 at 50 mg/kg body weight for 13 consecutive days and tumor volumes were monitored. Date are given as average with SEM (n = 4) and were compared by unpaired Student’s t test; *p<0.05. (B) Immunohistochemistry (IHC, upper panel) and immunoblot (lower panel) analysis of p-ERK1/2 levels in MRT allograft samples from mice treated with a single dose of vehicle or NVP-BGJ398 (50 mg/kg body weight) for 2 h. β-Tubulin expression was used to monitor equal loading.

Besides the *in vitro* analysis of FGFR-dependence in MRT cells, we aimed to test the effect of pharmacological FGFR inhibition on MRT growth *in vivo*. In line with the anti-proliferative effect of FGFR inhibition on G401 cells *in vitro*, we found that NVP-BGJ398 administration blocked tumor growth in a G401 xenograft transplantation model (Fig. S4). In order to investigate further the effect of FGFR inhibition in rhabdoid tumors, we utilized a murine SNF5 -deficient primary tumor, which was derived from a SNF5-heterozygous background upon spontaneous loss of heterozygosity (LOH) [29], [30], thus closely recapitulating MRT formation in humans. The murine-derived MRT model was histologically comparable to primary human SNF5-deficient tumors, consisting of SNF5-negative rhabdoid-like cells of variable size with enlarged nuclei and eosinophilic cytoplasm (data not shown). Treatment with NVP-BGJ398 significantly impaired growth of this MRT model in allograft transplantation studies (Fig. 6A). The growth inhibitory effect of FGFR inhibition was accompanied by a marked reduction of p-ERK1/2 levels in MRT tumor samples observed in immunohistological and immmunoblot analyses upon short-term, single dose NVP-BGJ398 treatment (Fig. 6B) These results indicate that pharmacological inhibition of FGFR signaling is able to suppress growth of MRTs in vivo.

### Elevated Expression of FGFR in Human Primary MRTs

**Figure 7:**
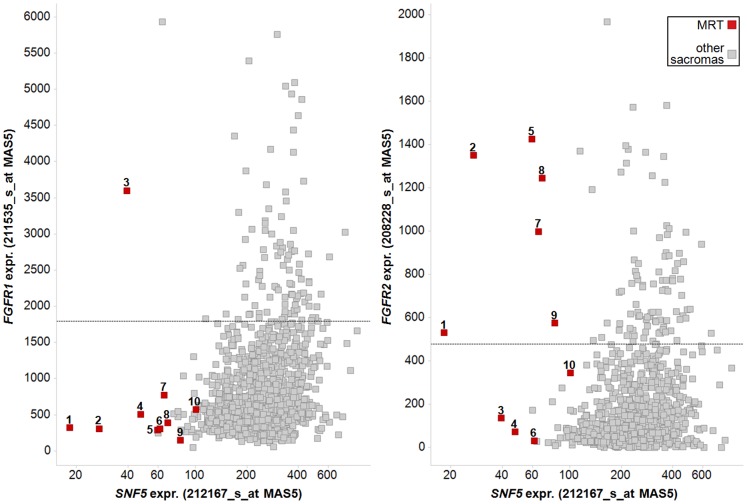
FGFR1 and FGFR2 expression in human primary MRTs. Scatter plot showing expression of *FGFR1* (left panel) and *FGFR2* (right panel) versus *SNF5* levels among human primary sarcoma samples. MRTs are indicated in red. Dashed line indicates threshold of 10^th^ percentile of highest *FGFR1* or *FGFR2* expression in sample set.

Our results point to a potential therapeutic use of FGFR inhibitors in the treatment of MRTs. We therefore investigated whether the SNF5/FGFR relationship is also observed in human MRT samples. For this purpose we interrogated *SNF5*, *FGFR1* and *FGFR2* mRNA expression in a total of 991 human primary soft tissue sarcomas from publically available Affymetrix datasets and including 10 rhabdoid tumors (File S2). This analysis revealed that the majority of SNF5-deleted rhabdoid tumors express elevated levels of *FGFR2* compared to the bulk of sarcoma samples. In addition, among the primary MRTs with low *FGFR2* expression one MRT sample (no. 3) displayed high levels of FGFR1 transcript (Fig. 7). This data suggests that upregulation of FGFR1 or FGFR2 occurs upon loss of SNF5 also in human MRTs and highlights the clinical relevance of our finding.

## Discussion

We have identified FGFR signaling as a novel deregulated pathway in SNF5-deficient MRTs and for the first time provide preclinical evidence that this tumor type can be therapeutically targeted by pharmacological inhibition of FGFRs. Furthermore, we show that in SNF5-expressing cells, the SWI/SNF complex has a repressive function on FGFR transcription, while re-expression of SNF5 in MRT cell lines abrogates FGFR expression. This indicates that loss of SNF5 leads to the upregulation of FGFR expression and pathway activation, which contributes to the highly malignant properties of MRTs.

MRTs are extremely aggressive pediatric tumors arising in very early childhood, which are often refractory to chemotherapy and fatal within the first year of diagnosis. Hence, there is tremendous need for novel clinical therapies for MRT patients. Inactivating bi-allelic mutations or deletions of SNF5 are found in nearly all MRTs [3], [5], [32], [33]. The critical function of SNF5 in rhabdoid tumor development is supported by knock-out mouse models of SNF5 showing development of lymphomas and MRTs with 100% penetrance and a median onset of only 11 weeks [30]. Besides genomic alterations leading to SNF5 loss of function, truncating deletions of BRG1 are present in a small subset of MRTs [34], further indicating that alterations in SWI/SNF function are causative for the development of this tumor type. However, other concomitant genomic aberrations are rarely found in MRTs. In fact, exome sequencing of a panel of 35 MRTs revealed that mutations or deletions of SNF5 are essentially the sole recurrent genomic alterations in MRTs [35]. Albeit, perturbation in SNF5 function causes genome-wide alterations in nucleosome positioning and global changes in transcription [14], [36]–[39]. This “epigenetic instability” might lead to the concurrent activation of multiple cancer-relevant pathways and account for the high malignancy of MRTs.

Indeed, besides the aberrant FGFR activity identified in this study, induction of several other known or potential oncogenes have been reported in MRTs. Most prominently, MRT lines depend on Hedgehog signaling mediated by over-expression of transcription factor GLI1 [14]. While elevated levels of *GLI1* are found in the absence of SNF5, re-expression of SNF5 in MRT lines strongly impaired *GLI1* expression, similar to the SNF5-dependent repression of FGFR transcription [14]. Likewise, the loss of SNF5 has been linked to the over-expression of Aurora A kinase and the Polycomb group protein EZH2 [19], [20]. Interestingly, in all cases SNF5 has been shown to be recruited to the respective target gene promoter region, indicating that SNF5 confers a direct repressive function on promoter activity. Similarly, we identified binding of SNF5 to the TSS of the *FGFR2* gene in the human fibroblast line BJ. Since siRNA-mediated knock-down of both SNF5 and the SWI/SNF ATPase subunit BRG1 led to increased expression of FGFR2, the repressive function of SNF5 potentially includes the recruitment of the SWI/SNF complex to specific target genes and corresponding changes in nucleosome architecture. Indeed, in the case of *GLI1*, the absence of SNF5 correlated with an open chromatin structure of the promoter region, while the TSS of the *GLI1* promoter was more densely packed with nucleosomes in the presence of SNF5 [14]. In addition, the SWI/SNF complex forms multiple interactions with histone modifying enzymes. For example, SNF5-dependent repression of cyclin D1 transcription involves the SWI/SNF-mediated recruitment of histone deacetylases (HDACs) to the promoter region [18]. Conversely, SNF5 is also able to activate gene expression, such as the tumor suppressor gene p16^INK4A^ [16], [17]. Here, the presence of SNF5 correlates with increased levels of trimethylated histone H3 lysine 4 (H3K4) at the *CDKN1A* locus [31]. In addition, via suppression of EZH2 transcription SNF5 confers an antagonistic function on Polycomb-mediated gene repression [20]. Thus, SNF5-mediated gene regulation is highly context specific and depends on the recruitment of regulatory cofactors.

In agreement with these reports, SNF5-dependent regulation of FGFR expression occurs in a context-specific fashion as well. While we observe elevated expression levels of FGFR1 in a subgroup of MRT cell lines and primary tumors, SNF5-deficiency correlates with induction of FGFR2 in another subset of MRT samples, indicating that cell-type specific mechanisms exist which confer susceptibility of either FGFR1 or FGFR2 to SNF5-dependent regulation. This is supported by our finding that inactivation of SNF5 in a panel of non-MRT sarcoma cell lines had no effect on FGFR expression, but led to increased FGFR2 levels in non-immortalized human fibroblasts, in line with the cell type specific activity of SNF5 on Aurora A kinase expression [19]. Furthermore, we did not observe increased expression levels of FGFR1 or FGFR2 in the atypical teratoid/rhabdoid tumor (AT/RT) cell line BT16 (data not shown), indicating that the correlation of SNF5-deficiency and elevated expression of FGFRs might be true only for non-CNS rhabdoid tumors. Interestingly, SNF5 has been recently implicated in the development of familial schwannomatosis at high frequency [40]–[43] and is inactivated in a couple of tumor types besides MRTs. For instance, homozygous deletions of the SNF5 locus 22q11.2 are found in small-cell hepatoblastomas and poorly differentiated chordomas [44], [45] and inactivating mutations and/or deletion of SNF5 are observed in extraskeletal myxoid chondrosarcomas [46], undifferentiated sarcomas [47], epitheliod sarcomas [48] and meningiomas [41], [49]. It will be interesting to investigate in future studies whether FGFR signaling is up-regulated in these tumor types as well. Reduced expression of SNF5 is also observed in synovial sarcoma [50], [51], and, interestingly, FGFR pathway inhibition exerts anti-proliferative effects in synovial sarcoma models [52]. Upregulation of *FGFR2* in synovial sarcoma has been linked to the synovial sarcoma-associated oncogene SYT–SSX2 [53]. Still, it might be worth to investigate the effect of reduced SNF5 expression on FGFR pathway activity in synovial sarcoma.

The findings reported in this study show for the first time that targeted inhibition of FGFRs in SNF5-deleted MRTs may be a beneficial therapeutic modality for this cancer type. In support of this strategy, we have identified human primary MRTs that show concomitant loss of SNF5 and elevated FGFR1 or FGFR2 mRNA levels. However, while we find strong anti-proliferative effects of FGFR inhibitor treatment on MRT cell line and murine allograft models, further studies are necessary to determine whether specific combination therapies with FGFR targeting agents may be more beneficial, since multiple oncogenic pathways are aberrantly activated in MRTs. In this regard, it may be of interest to test drug combinations in future studies, e. g. using inhibitors to FGFRs, Aurora A kinase and CDK4, which might lead the way to an effective clinical therapy for this lethal pediatric cancer.

## Supporting Information

Figure S1 **FGFR inhibition with NVP-BGJ398 impairs growth of MRT cell lines** ***in vitro*** **.** Proliferation assays with NVP-BGJ398 in A204 (A), G401 (B) and G402 (C) cells. Cell were plated in 96-wells and treated with NVP-BGJ398 at the indicated concentrations for 4 d. The effect on proliferation was assayed by methylene blue staining. Half maximal inhibitory concentrations (IC_50_) for NVP-BGJ398 were calculated using XLfit and are indicated in the graphs.
(TIF)

Figure S2 **FGFR1 and FGFR2 expression and copy number among the CCLE dataset.** Scatter plot showing expression and copy number levels for *FGFR1* (left panel) and *FGFR2* (right panel) within the CCLE. MRT lines A204, G401 and G402 are indicated in red.
(TIF)

Figure S3 **Re-expression of SNF5 in SNF5-deficient cell lines abrogates FGFR expression.** Effect of SNF5 re-expression on FGFR1 levels in MRT line A204 (A), KYM1 rhabdomyosarcoma cells (B) and on FGFR2 expression in HLC1 lung adenocarcinoma cells (C). Protein expression was analyzed by immunoblot five days post retroviral transduction of SNF5. β-Tubulin expression was used to monitor equal loading. (D) SNF5 expression and copy number among the CCLE dataset. (E) FGFR1 and (F) FGFR2 expression versus SNF5 expression among the CCLE dataset. MRT lines A204, G401 and G402 are indicated in red.
(TIF)

Figure S4 **FGFR inhibition by NVP-BGJ398 impairs growth of an MRT xenograft model** ***in vivo*** **.** G401 MRT cells were grown subcutaneously in nude mice. Treatment with NVP-BGJ398 at 50 mg/kg body weight started when tumor volume reached at least 150 mm^3^. Mice were treated daily for 24 days. Tumor volume changes over the course of treatment are shown as average with SEM (n≥7). Statistical analysis was performed by unpaired Student’s t test with respect to vehicle-treated controls (*p<0.05).
(TIF)

Materials and Methods S1 (DOCX)

File S1 **NVP-BGJ398 proliferation assays.**
(XLSX)

File S2 **Primary sarcoma expression data.**
(XLSX)
