# Supplementary figures and images for: Fibroblast Growth Factor Receptors as Novel Therapeutic Targets in SNF5-Deleted Malignant Rhabdoid Tumors
Source: PLoS One. 2013 Oct 30;8(10):e77652. doi: 10.1371/journal.pone.0077652 (PMC3813701; doi:10.1371/journal.pone.0077652)

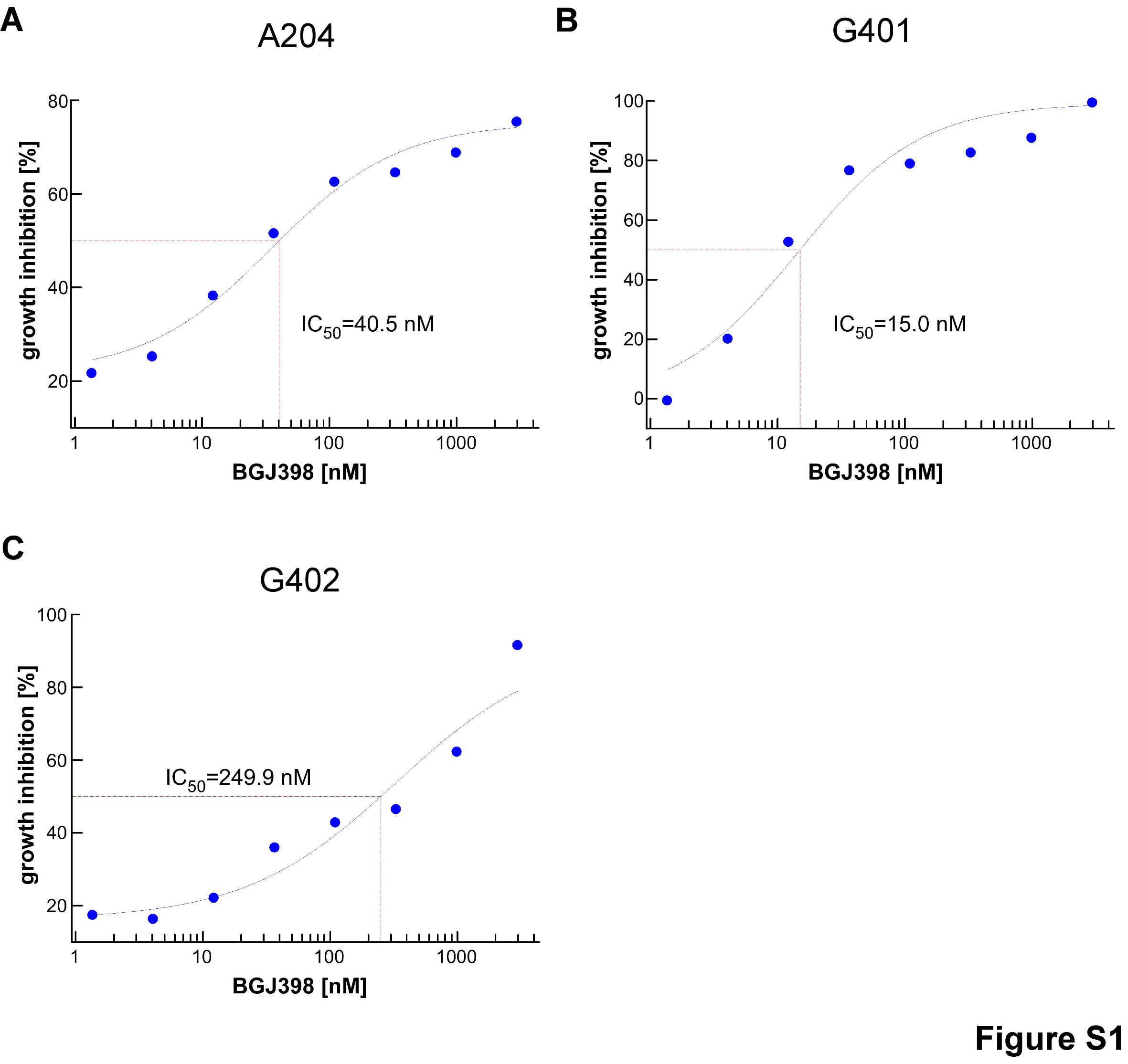

Supplement: Figure S1 — FGFR inhibition with NVP-BGJ398 impairs growth of MRT cell lines in vitro . Proliferation assays with NVP-BGJ398 in A204 (A), G401 (B) and G402 (C) cells. Cell were plated in 96-wells and treated with NVP-BGJ398 at the indicated concentrations for 4 d. The effect on proliferation was assayed by methylene blue staining. Half maximal inhibitory concentrations (IC50) for NVP-BGJ398 were calculated using XLfit and are indicated in the graphs. (TIF) [file pone.0077652.s001.tif]

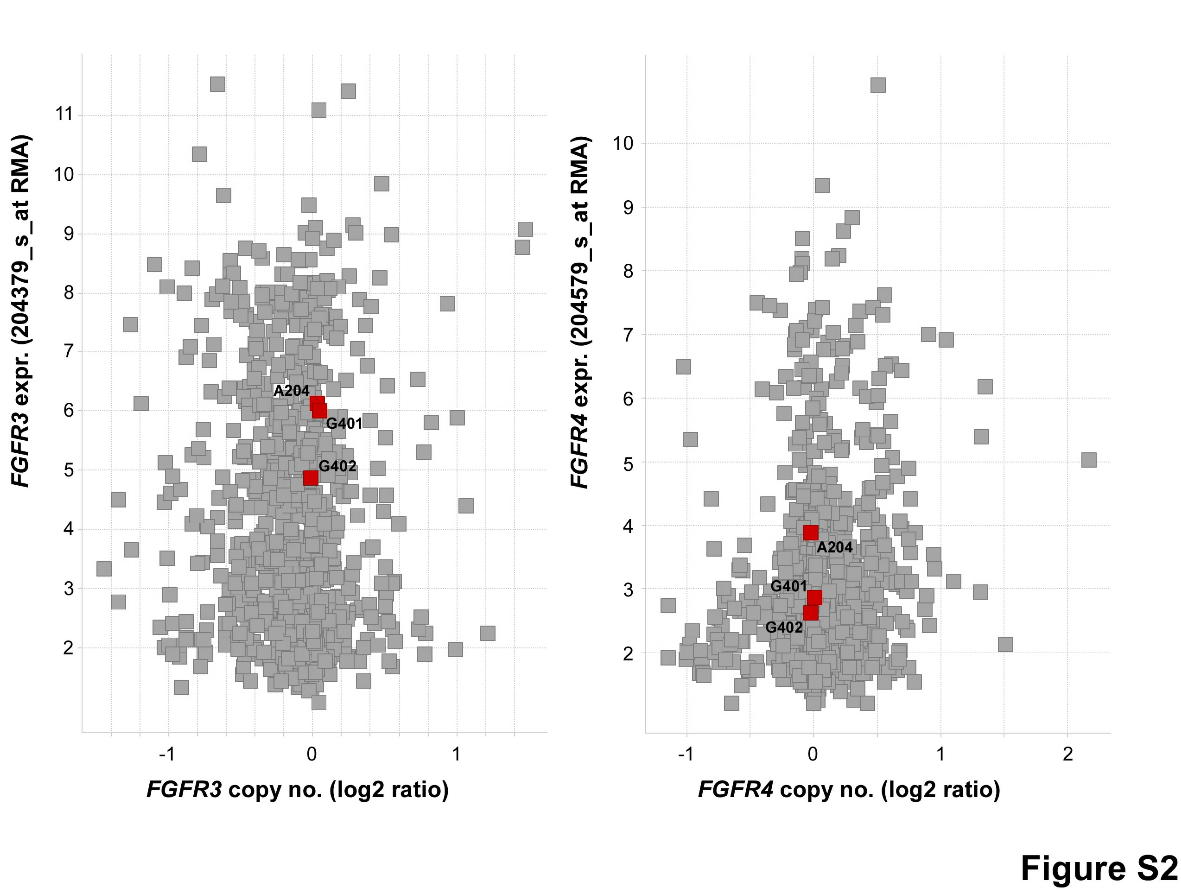

Supplement: Figure S2 — FGFR1 and FGFR2 expression and copy number among the CCLE dataset. Scatter plot showing expression and copy number levels for FGFR1 (left panel) and FGFR2 (right panel) within the CCLE. MRT lines A204, G401 and G402 are indicated in red. (TIF) [file pone.0077652.s002.tif]

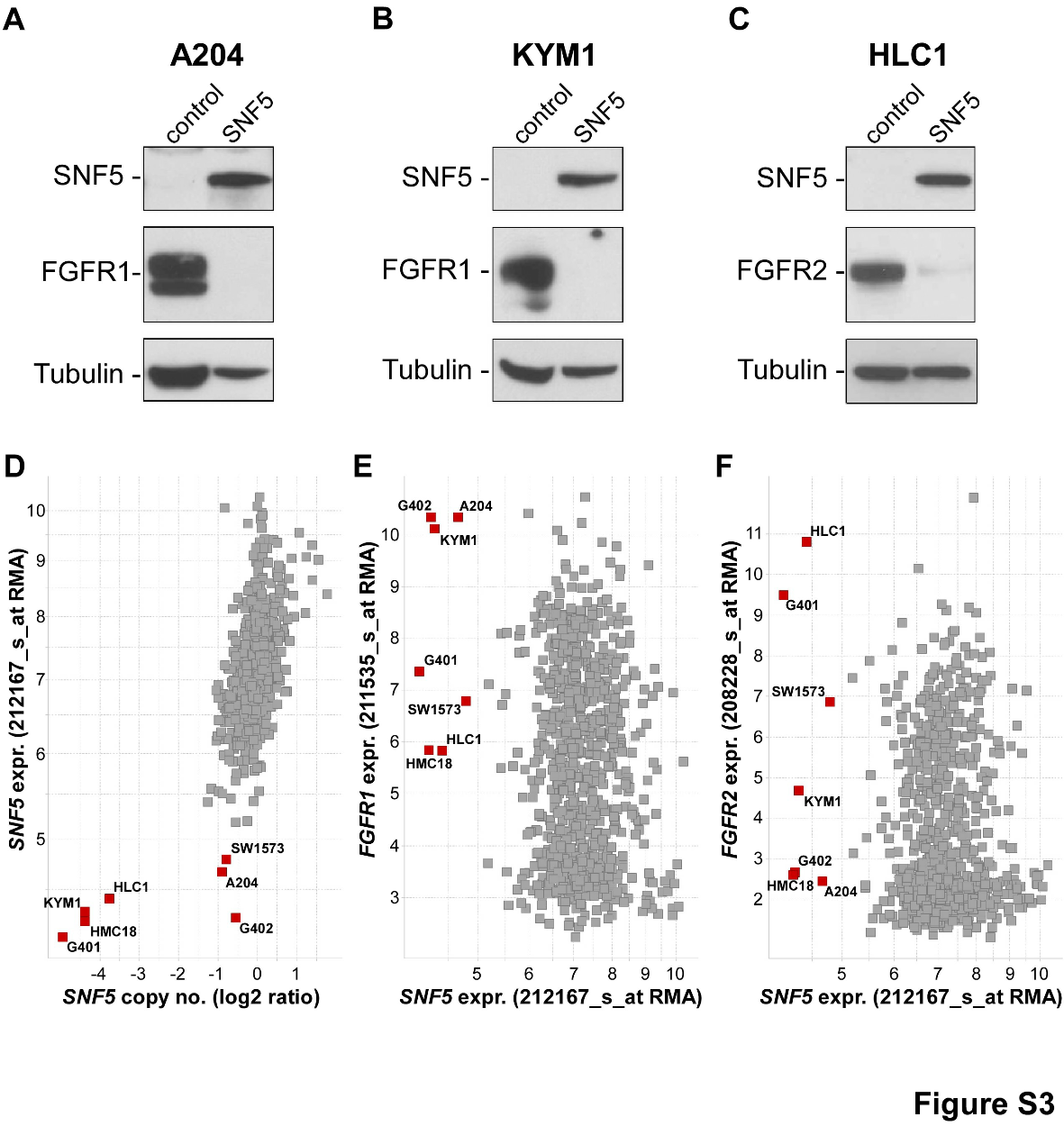

Supplement: Figure S3 — Re-expression of SNF5 in SNF5-deficient cell lines abrogates FGFR expression. Effect of SNF5 re-expression on FGFR1 levels in MRT line A204 (A), KYM1 rhabdomyosarcoma cells (B) and on FGFR2 expression in HLC1 lung adenocarcinoma cells (C). Protein expression was analyzed by immunoblot five days post retroviral transduction of SNF5. β-Tubulin expression was used to monitor equal loading. (D) SNF5 expression and copy number among the CCLE dataset. (E) FGFR1 and (F) FGFR2 expression versus SNF5 expression among the CCLE dataset. MRT lines A204, G401 and G402 are indicated in red. (TIF) [file pone.0077652.s003.tif]

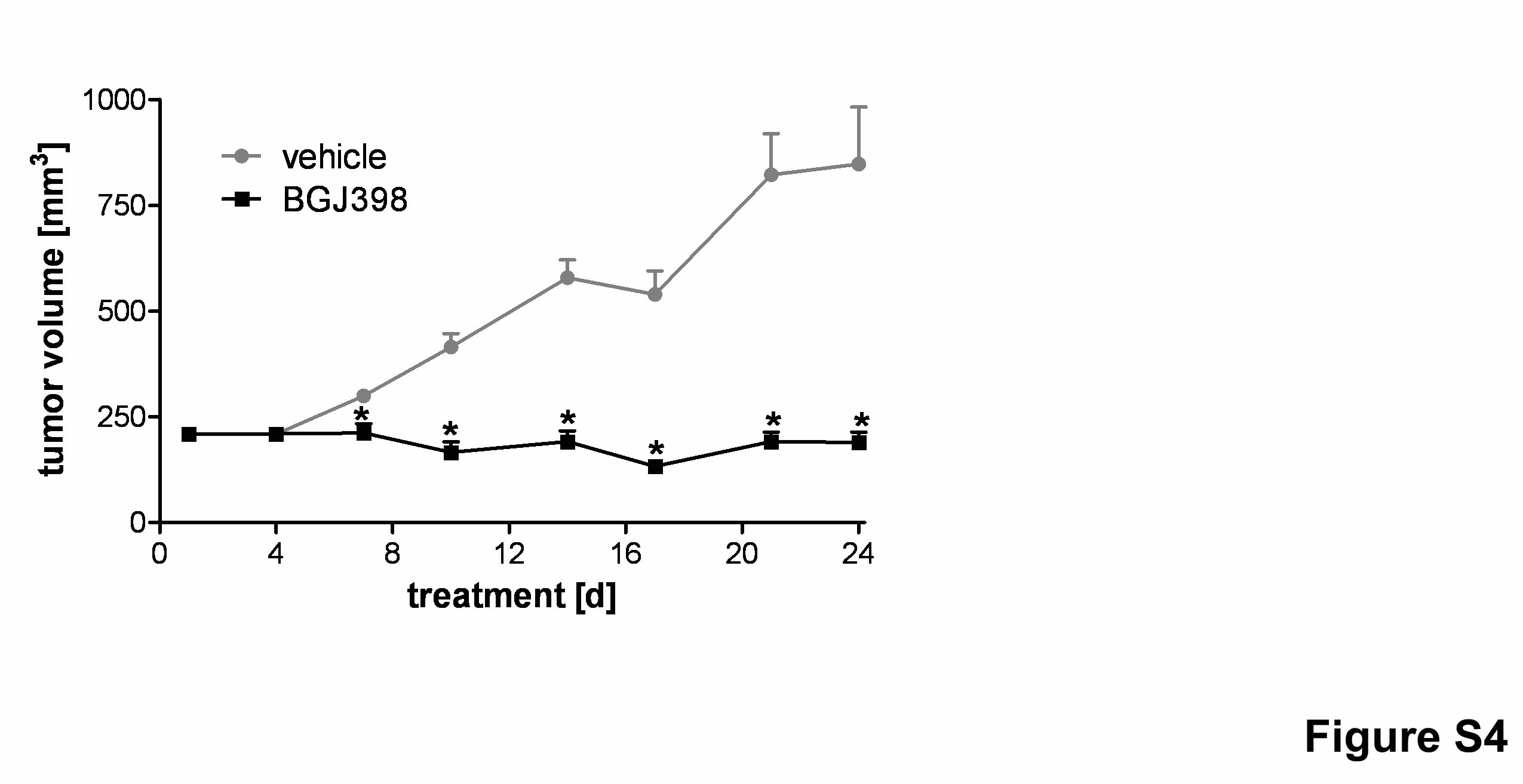

Supplement: Figure S4 — FGFR inhibition by NVP-BGJ398 impairs growth of an MRT xenograft model in vivo . G401 MRT cells were grown subcutaneously in nude mice. Treatment with NVP-BGJ398 at 50 mg/kg body weight started when tumor volume reached at least 150 mm3. Mice were treated daily for 24 days. Tumor volume changes over the course of treatment are shown as average with SEM (n≥7). Statistical analysis was performed by unpaired Student’s t test with respect to vehicle-treated controls (*p<0.05). (TIF) [file pone.0077652.s004.tif]
