# Supplementary material for: Fibroblast Growth Factor Receptors as Novel Therapeutic Targets in SNF5-Deleted Malignant Rhabdoid Tumors
Source: PLoS One. 2013 Oct 30;8(10):e77652. doi: 10.1371/journal.pone.0077652 (PMC3813701; doi:10.1371/journal.pone.0077652)
Supplement: Materials and Methods S1 — (DOCX) [file pone.0077652.s005.docx]

**Supplemental Material and Methods**

**Cell lines**

HLC1 were obtained from RIKEN and maintained in HAM’s F12K with stable Glutamine and 10% FCS (Hyclone). KYM1 cells were obtained from HSRRB and maintained in DMEM/HAM's F12K (Amimed) with 10 % FCS and 2 mM L-glutamine.

**Global gene expression and copy number visualization**

The publicly available CCLE dataset (26) was utilized for gene expression and copy number analysis across a panel of approximately 1000 cancer cell lines. Probesets analyzed were 204379_s_at for FGFR3, 204579_s_at for FGFR4 and 212167_s_at for SNF5. Data was visualized using Spotfire (TIBCO).

**Proliferation assays**

For manual cell proliferation assays, cells were seeded in 96-well plates at a density of 10^3^ to 10^4^ cell per well in a volume of 100 µl. Media containing dilutions of NVP-BGJ398 or DMSO was added 24 hours thereafter. After 4 days cell density was analyzed using methylene blue staining. The concentration of compound providing 50% of proliferation inhibition (IC_50_) was determined using XLfit (idbs).

**Xenograft mouse models and anti-tumor efficacy studies**

Tumor cells were implanted subcutaneously into female nude mice (*nu*/*nu*, Harlan) at 8 weeks of age. Trypsinized G401 cell monolayers were washed and suspended at 5 x 10^7^ cells/ml in cold phosphate-buffered saline with 50% Matrigel (BD Biosciences). Each animal was inoculated subcutaneously in the right flank with 0.2 ml of the suspension (1 x 10^7^ cells). The tumors were periodically callipered in two dimensions to monitor growth as the mean volume approached 160–230 mm^3^. Eight days after tumor cell implantation, on day 1 of the study, the animals were sorted into two groups of ten mice, with treatment with NVP-BGJ398 or vehicle control was initiated. Treatment was performed once daily by oral gavage. Tumor volumes were monitored at the indicated times over the course of treatment. Tumor size, in mm^3^, was calculated from: Tumor Volume = 2 / w^2^ × l; where w = width and l = length, in mm, of the tumor.
